# Cancer Risk in Patients Treated with the JAK Inhibitor Tofacitinib: Systematic Review and Meta-Analysis

**DOI:** 10.3390/cancers15082197

**Published:** 2023-04-07

**Authors:** Cristina Bezzio, Marta Vernero, Davide Giuseppe Ribaldone, Eleonora Alimenti, Gianpiero Manes, Simone Saibeni

**Affiliations:** 1 IBD Center, Gastroenterology Unit, Rho Hospital, ASST Rhodense, 20017 Rho, Italy; 2 Department of Medical Sciences, University of Turin, 10124 Turin, Italy; 3 Department of Medical Sciences, University of Pavia, 27100 Pavia, Italy

**Keywords:** tofacitinib, drug safety, malignancy risk, IMIDs, biologics, therapy, ulcerative colitis, rheumatoid arthritis

## Abstract

**Simple Summary:**

Tofacitinib is a relatively novel therapy for immune-mediated inflammatory diseases, including rheumatoid arthritis, psoriatic arthritis, and ulcerative colitis. It is a small-molecule drug that exerts its effects by inhibiting Janus kinases. Recently, concerns have been raised about the drug’s safety in terms of cardiovascular side effects and cancer risk. This meta-analysis determined the risk of cancer in patients treated with tofacitinib for different clinical indications, compared to both a placebo and other therapies. We did not find any difference in the cancer risk between tofacitinib and either the placebo or biological drugs overall. In contrast, we found only a slightly higher risk of cancer in patients treated with tofacitinib compared with the patients treated with drugs that inhibit the tumor necrosis factor. Therefore, further studies are needed to better define the cancer risk of tofacitinib therapy.

**Abstract:**

Tofacitinib is approved for several immune-mediated inflammatory diseases, but safety concerns have recently been raised. We searched PubMed (accessed on 27 February 2023) for original articles regarding tofacitinib’s cancer risk when used for rheumatoid arthritis, ulcerative colitis, Crohn’s disease, psoriatic arthritis, and ankylosing spondylitis. Of the 2047 initial records, 22 articles describing 26 controlled studies (including 22 randomized controlled trials) were selected. In the comparison between tofacitinib and any control treatment, the relative risk (RR) for any cancer was 1.06 (95% CI, 0.86–1.31; *p* = 0.95). In separate comparisons between tofacitinib and either a placebo or biological therapy, no difference was found in the overall cancer risk (vs. placebo, RR = 1.04; 95% CI, 0.44–2.48; *p* = 0.95; vs. biological drugs, RR = 1.06; 95% CI, 0.86–1.31; *p* = 0.58). When tofacitinib was compared to tumor necrosis factor (TNF) inhibitors, the overall cancer RR was 1.40 (95% CI, 1.06–2.08; *p* = 0.02). Similarly, significant results were obtained for all cancers, except for non-melanoma skin cancer (RR = 1.47; 95% CI, 1.05–2.06; *p* = 0.03), and for this skin cancer alone (RR = 1.30; 95% CI, 0.22–5.83; *p* = 0.88). In conclusion, no difference in the overall cancer risk was found between tofacitinib and either a placebo or biological drugs, while a slightly higher risk was found in patients treated with tofacitinib than anti-TNF agents. Further studies are needed to better define the cancer risk of tofacitinib therapy.

## 1. Introduction

Tofacitinib is one of several Janus kinase (JAK) inhibitors that form a novel class of drugs with immunomodulatory and anti-inflammatory effects and are widely prescribed for several immune-mediated inflammatory diseases (IMIDs) [1,2]. JAKs are intracellular, non-receptor tyrosine kinases that convert extracellular signals into a wide range of cellular responses. JAKs, together with signal transducer and activator of transcription proteins (STATs) and other proteins, form the intracellular JAK/STAT signaling pathway. STAT proteins bind to DNA and regulate the transcription of proteins required for key physiological processes, including immunity and inflammation [3,4]. Aberrant JAK/STAT signaling is implicated in the pathogenesis of several IMIDs [5].

Tofacitinib was first approved for the treatment of rheumatoid arthritis by the US Food and Drug Administration (FDA) in 2012 [6]. Soon after, it was also approved for psoriatic arthritis (PsA), ankylosing spondylitis (AS), ulcerative colitis (UC), and polyarticular juvenile idiopathic arthritis [7]. The drug has been under investigation as an immunosuppressor for solid organ transplantation [8], and recently, it has been suggested for use in immunomodulated dermatological diseases, such as *Pyoderma gangrenosum* [9,10,11,12].

Despite tofacitinib’s efficacy in several IMIDs, major concerns about its safety have recently been raised. Some authors suggested that tofacitinib could be associated with increased risks of major adverse cardiovascular events, infections, blood clots, cancer (namely lung and skin cancers), and death [13,14,15]. As a result, the FDA released a safety communication about these risks [16].

The risk of cancer after treatment with tofacitinib has been assessed in many studies, with discordant results. For example, there was a higher risk of cancer in patients who took tofacitinib than an anti-tumor necrosis factor (TNF) agent after 18 months of therapy for rheumatoid arthritis, according to a post-hoc analysis [17] of a randomized controlled trial (ORAL SURVEILLANCE) [15]. The most common malignancy was lung cancer, for which the risk was higher in patients who received 10 mg (an off-label dosage in rheumatoid arthritis) of tofacitinib twice daily than in control patients. However, we have to underline that the ORAL Surveillance trial was a non-inferiority trial designed to assess the safety of tofacitinib compared with a TNF inhibitor in RA-active patients, despite methotrexate treatment, aged higher than 50 years and with at least one additional cardiovascular risk factor. In particular, this trial evaluated the hypothesis that the risk of major adverse cardiovascular events or cancers, excluding non-melanoma skin cancer, would not be at least 1.8 times higher with tofacitinib (combined doses of 5 mg and 10 mg twice daily) than with a TNF inhibitor. [15].

In contrast, a study of a large cohort of RA patients in routine care did not find an increased risk of any malignancy associated with tofacitinib therapy compared with anti-TNF treatment [18]. Given the heterogeneity of the evidence on this topic, this meta-analysis investigated the risk of cancer in patients who took tofacitinib compared to a placebo or another therapy, in the settings of both randomized controlled trials and clinical practice.

## 2. Materials and Methods

The present systematic review with meta-analysis was previously registered in the PRISMA database and was conducted following PRISMA reporting guidelines.

### 2.1. Bibliographic Research and Article Selection

We analyzed original research articles published in English about tofacitinib and cancer or about tofacitinib and its use for approved or experimental indications. Relevant articles were first identified by searching PubMed.gov (“All Databases”) with the following search strings: “tofacitinib AND cancer”; “tofacitinib AND ulcerative colitis”; “tofacitinib AND Crohn”; “tofacitinib AND rheumatoid arthritis”; “tofacitinib AND psoriatic arthritis”; “tofacitinib AND ankylosing spondylitis”; and “tofacitinib AND juvenile idiopathic arthritis”. The bibliographic search was completed on 10 October 2022.

Three authors (D.G.R., M.V. and E.A.) independently reviewed the titles and abstracts of the articles identified by the bibliographic research and selected potentially relevant studies. The same authors read the full texts of the initially selected papers to check the inclusion and exclusion criteria. Inclusion criteria were as follows: (1) research comparing (as a primary or secondary outcome) the incidence of malignancies between patients treated with tofacitinib and controls (i.e., treated with another therapy or placebo); (2) original papers reporting a clinical trial, cohort study or observational study; and (3) studies in the English language. Exclusion criteria were as follows: (1) the lack of a control group; (2) lack of comparability in baseline characteristics between case and control groups or between different arms of a clinical trial; (3) papers reporting individual cases, meta-analyses, pooled analyses, or analyses of data from other original papers; (4) reviews; and (5) studies conducted on children. Divergences in opinion among the three reviewers were solved by discussion until an agreement was obtained or, when a consensus was not reached, a fourth author (S.S.) was consulted. Finally, reference lists of articles selected for the analysis were screened for other titles that met the study’s inclusion and exclusion criteria.

### 2.2. Data Extraction and Quality Assessment

For each paper included in the analysis, the following information was collected: number of patients in the different study groups, mean follow-up time (in months), number of cancers in the different groups, and types of cancers (when available). For articles with more than one control group or more than one timepoint of follow-up, we divided the data into two or more parts and treated the parts as separate studies in the analysis.

As different indications and different phases trials required different doses of tofacitinib in the treatment group (and the dosage frequently varied during the study), the dosage of the drug was not taken into account for the statistical analysis.

The included articles were evaluated using the Newcastle–Ottawa Scale (NOS), which judges study quality according to the following three perspectives: selection of the study groups, comparability of the groups, and ascertainment of the exposure of interest.

### 2.3. Meta-Analyses

For the meta-analyses, we expressed the follow-up duration of each study in units of person-months and used this value as the denominator for calculating risks, thereby reducing the heterogeneity in follow-up time among the studies. When the number of malignancies was zero in all study groups, the study was removed from the statistical analysis because the relative risk was not calculable. The overall risk of malignancy was calculated, as were the risks for all cancers, except non-melanoma skin cancer (NMSC) and for NMSC alone. When the type of tumor was gynecological, only the female study population was taken into account for the risk calculation.

To identify publication bias, funnel plots were created and visually evaluated. A symmetrical inverted funnel was taken to indicate the absence of publication bias, while asymmetry was interpreted as publication bias [19].

Statistical analysis was conducted using Med Calc software (version 18.9.1; Ostend, Belgium). Cochran’s Q and I^2^ statistics were used to estimate heterogeneity across the studies. When heterogeneity was observed (Q-test *p* < 0.05, I^2^ > 50%), a random-effects model was used; otherwise, a fixed-effect model was employed. A *p* value < 0.05 was treated as statistically significant.

## 3. Results

**Figure 1:**
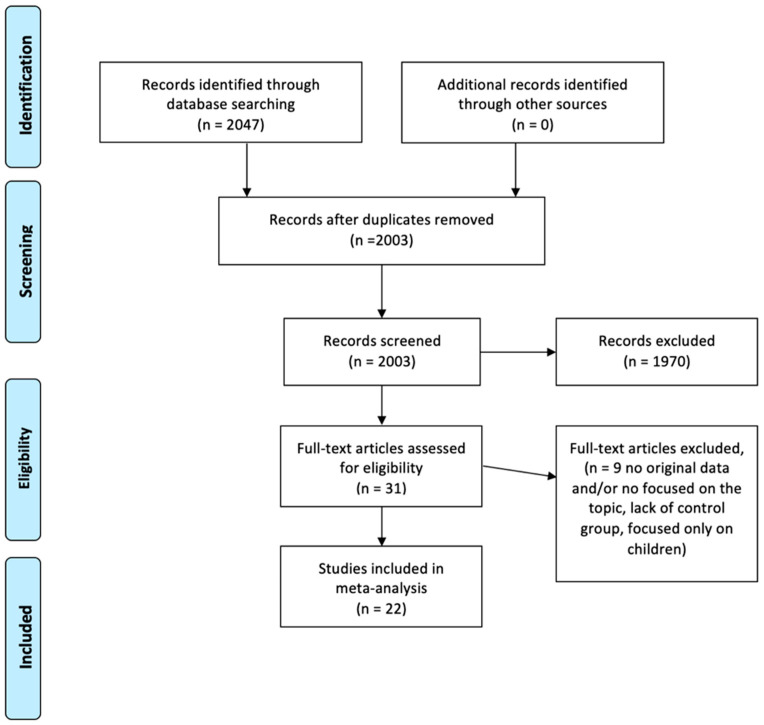
PRISMA flowchart of screened and included articles.

Our bibliographic searches gave a total of 2047 results (Figure 1). After duplicates and non-pertinent titles were eliminated, 31 full texts were available for consideration. After reading these articles, we excluded nine for not meeting all the inclusion criteria or for meeting one or more of the exclusion criteria. Thus, 22 articles were included in the study.

The 22 selected articles had been published between 2011 and 2022 and reported on clinical trials (18 articles) and observational studies (Table 1). The pathology most often investigated was rheumatoid arthritis (13 articles), followed by ulcerative colitis (5 articles); the other articles investigated ankylosing spondylitis, Crohn’s disease, and psoriatic arthritis, while no article on juvenile idiopathic arthritis was included, as this is a pediatric disease and one of the exclusion criteria were the pediatric population. Two of the articles reported on more than one clinical trial, and one article described a study with two control groups. Thus, we had 26 separate studies for evaluation.

The quality of the included studies was assessed at the article level, resulting in a mean NOS score of 8.5 (Table 2). Thirteen studies had detected one or more cancer cases during follow-up (Table 3). Thus, these 13 studies were included in the meta-analyses, with a mean of 319.98 months per person of follow up for the treatment group and 255.29 for the control group.

**Figure 2:**
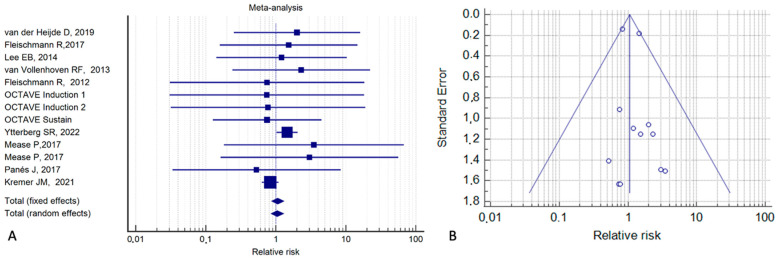
Risk of malignancy in tofacitinib-treated patients vs. control patients who received a placebo or an active treatment (**A**). Corresponding funnel plot (**B**) [2,15,20,21,22,24,25,36,39].

In the meta-analysis, we first assessed the overall risk of malignancy in patients who had been treated with tofacitinib and in control patients who had received a placebo or an active treatment. As shown in the forest plot (Figure 2A), the relative risk (RR) from a fixed-effect model was 1.06 (95% CI, 0.86–1.31; *p* = 0.57; I^2^ = 0%). The corresponding funnel plot (Figure 2B) was symmetrical, confirming that the publication bias was low.

**Figure 3:**
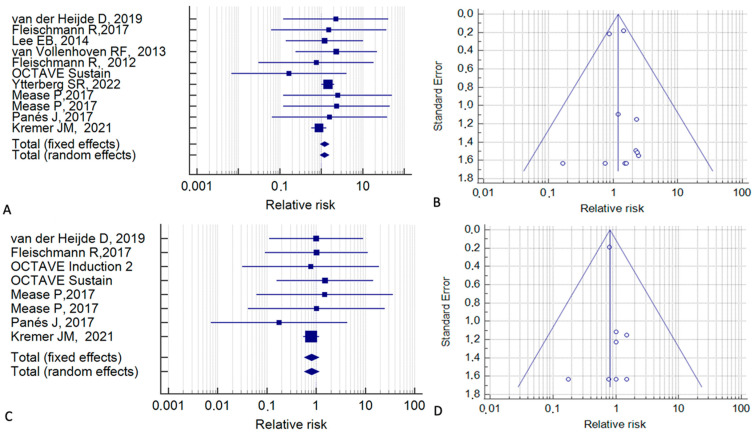
Risk of malignancy, treating non-melanoma skin cancer separately, in tofacitinib-treated patients vs. control patients who received a placebo or an active treatment. (**A**,**B**) Malignancy excluding non-melanoma skin cancer. (**C**,**D**) Non-melanoma skin cancer alone [2,15,20,21,22,24,25,35,36,39].

We repeated the meta-analysis for all cancers excluding NMSC using data from 11 studies (Figure 3A). From the fixed-effect model, it can be observed that the RR for these cancers was 1.20 (95% CI, 0.93–1.55; *p* = 0.15; I^2^ = 0%), indicating that the neoplastic risk is not increased with tofacitinib.

For NMSC, data from eight studies were available. For this cancer, the fixed-effect model gave an RR of 0.80 (95% CI, 0.57–1.15; *p* = 0.23; I^2^ = 0%) (Figure 3C). The corresponding funnel plots (Figure 3B,D) were symmetrical.

**Figure 4:**
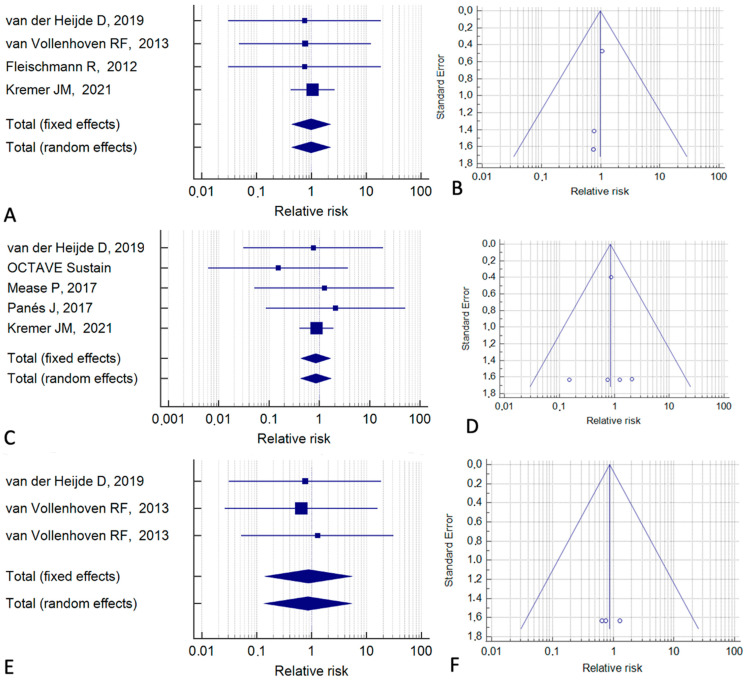
Meta-analyses for different types of cancer. (**A**,**B**) Lung cancer. (**C**,**D**) Breast cancer. (**E**,**F**) Cervical cancer [2,20,24,25,35,36,39].

We next examined the risk of individual types of cancer (considering the patients given a placebo or an active treatment as the control group). Data were available and sufficient for statistical analysis regarding lung cancer, breast cancer, and cervical cancer (Figure 4). For lung cancer, the fixed-effect model gave an RR of 0.98 (95% CI, 0.44–2.23; *p* = 0.97; I^2^ = 0%) (Figure 4A). For breast cancer, the RR was 0.84 (95% CI, 0.43–1.68; *p* = 0.63; I^2^ = 0%) (Figure 4C) and for cervical cancer, the RR was 0.88 (95% CI, 0.14–5.48; *p* = 0.88; I^2^ = 0%) (Figure 4E). These results indicate that the individual neoplastic risks are not increased with tofacitinib. Figure 4B,D,F show relative Funnel plots.

**Figure 5:**
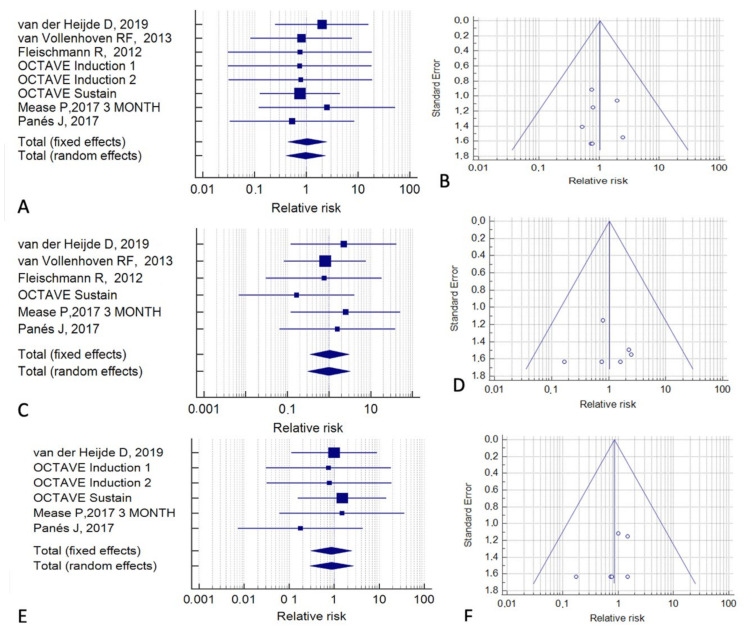
Risk of malignancy in tofacitinib-treated patients relative to placebo-treated patients. (**A**,**B**) All cancers. (**C**,**D**) All cancers except non-melanoma skin cancer. (**E**,**F**) Non-melanoma skin cancer. (**A**,**C**,**E**) Forest plots of relative risk. (**B**,**D**,**F**) Funnel plots for the assessment of publication bias [2,20,24,25,35,36,39].

In other analyses, we distinguished the included studies according to the type of control group. Among the eight studies with a placebo control (Figure 5), no difference was found in the overall cancer risk between tofacitinib and the placebo (RR = 1.04; 95% CI, 0.44–2.48; *p* = 0.95; I^2^ = 0%) (Figure 5A,B). Similar results were found for all cancers, excluding NMSC (RR = 1.03; 95% CI, 0.35–3.06; *p* = 0.95; I^2^ = 0%) (Figure 5C,D) and for NMSC alone (RR = 0.86; 95% CI, 0.29–2.49; *p* = 0.70; I^2^ = 0%) (Figure 5E,F).

**Figure 6:**
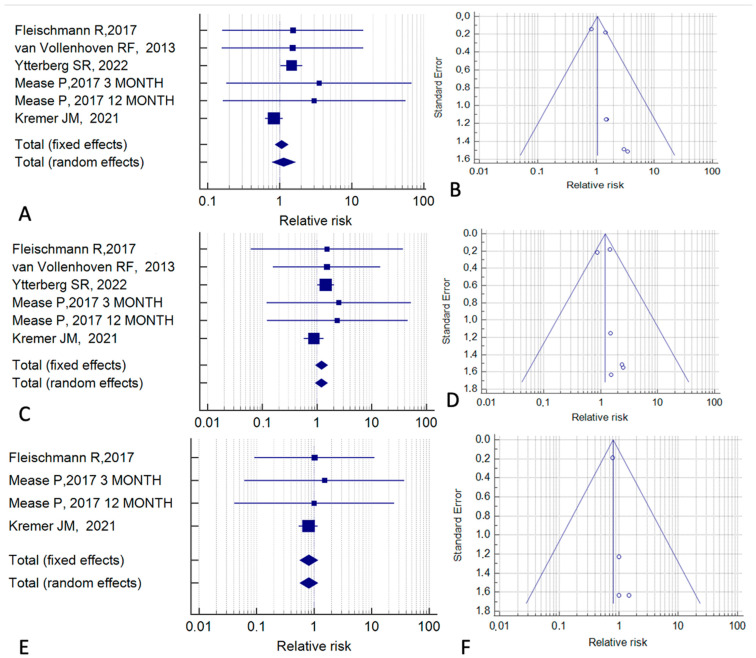
Cancer risk associated with tofacitinib treatment relative to treatment with biological drugs. (**A**,**B**) All cancers. (**C**,**D**) All cancers except non-melanoma skin cancer. (**E**,**F**) Non-melanoma skin cancer. (**A**,**C**,**E**) Forest plots of relative risk. (**B**,**D**,**F**) Funnel plots for the assessment of publication bias [15,21,24,35,39].

Next, we considered the six studies whose control group received biological therapy (Figure 6). In a fixed-effect model, the overall RR for cancer was 1.06 (95% CI, 0.86–1.31; *p* = 0.58; I^2^ = 31.18%) (Figure 6A,B). For all cancers except NMSC, the RR was 1.20 (95% CI, 0.93–1.57; *p* = 0.16; I^2^ = 0%) (Figure 6C,D). For NMSC, the RR was 0.89 (95% CI, 0.56–1.17; *p* = 0.25; I^2^ = 0%) (Figure 5E,F).

**Figure 7:**
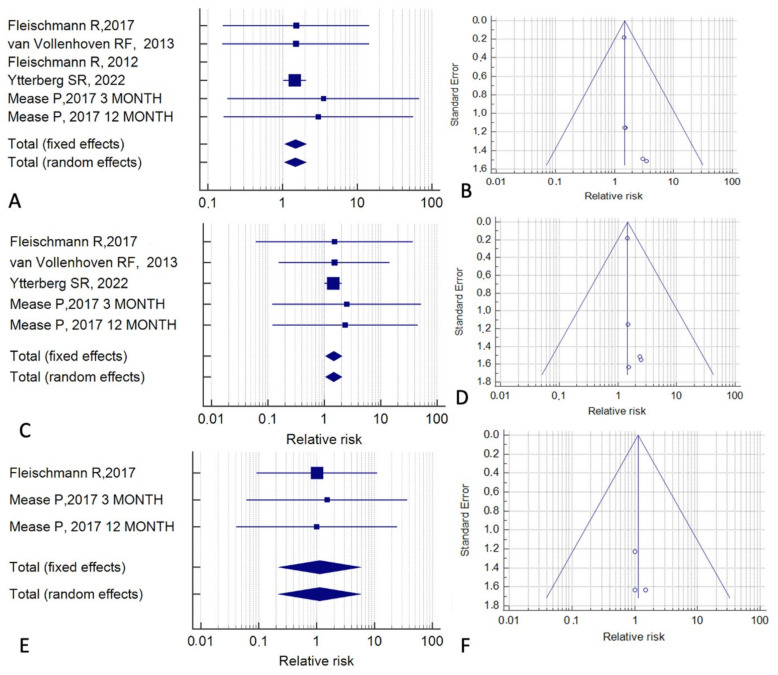
Cancer risk associated with tofacitinib treatment relative to treatment with anti-TNF agents. (**A**,**B**) All cancers. (**C**,**D**) All cancers except non-melanoma skin cancer. (**E**,**F**) Non-melanoma skin cancer. (**A**,**C**,**E**) Forest plots of relative risk. (**B**,**D**,**F**) Funnel plots for the assessment of publication bias [15,21,24,25,35,39].

Finally, we evaluated the six studies that had anti-TNF agents as the control treatment (Figure 7). In a fixed-effect model, the RR for overall cancer was 1.40 (95% CI, 1.07–2.09; *p* = 0.02; I^2^ = 0%). This finding indicates that the relative risk of malignancy is increased in patients treated with tofacitinib, in comparison with patients treated with anti-TNF agents (Figure 7A,B). A similar result was obtained for the risk of all cancers excluding NMSC (RR = 1.47; 95% CI, 1.05–2.07; *p* = 0.03; I^2^ = 0%) (Figure 7C,D). In contrast, no significant effect was observed in the analysis of NMSC alone (RR = 1.30; 95% CI, 0.22–5.84; *p* = 0.88; I^2^ = 0%) (Figure 6E,F).

Finally, when sufficient data were available, we analyzed the cancer risk in each indication subgroup. As reported in the Supplementary Materials, this was only possible for RA, SpA and UC. The majority of the included studies investigated RA patients; among these studies, no significant risk was found for the overall cancer risk, NMSC, or lung cancer in patients undergoing tofacitinib vs. the control group including a placebo and other biological drugs, the placebo only, or biological drugs. Conversely, the overall cancer risk was slightly higher in patients who had taken tofacitinib vs. anti-TNF agents (RR 1.43; 95% CI 1.03–2.04; I^2^ = 0.00%; *p* = 0.03) (Supplementary Materials, Figures S1 and S2). No significantly higher risk was found in SpA or UC patients who had taken tofacitinib vs. the control group (Supplementary Materials, Figures S3 and S4).

## 4. Discussion

Our meta-analysis assessed the risk of cancer in patients treated with tofacitinib compared to control patients who received a placebo or an active IMID treatment. Our study identified 26 studies on the use of tofacitinib for an IMID in twenty-two randomized controlled trials and four observational studies. The pathology most often investigated was rheumatoid arthritis, but there also were studies on ulcerative colitis, ankylosing spondylitis, Crohn’s disease, and psoriatic arthritis. At least one cancer was reported during follow-up in 13 studies, which were included in our meta-analysis.

Despite the plenitude of published papers on the efficacy and safety of tofacitinib in rheumatological, dermatological, and gastroenterological diseases, few of these studies had a control group. Indeed, only 22 of the initial 2047 articles retrieved from PubMed were included in our study. Moreover, even though malignancy was a safety issue for all of the 26 included studies, in 13 of these studies, no case of cancer was detected, so they were excluded from the meta-analysis because the relative risk was not calculable. The overall quality of the included articles was high (mean NOS score: 8.5) and the heterogeneity of data was low, allowing us to use a fixed-effect model in all of the analyses. The high quality was probably due to the fact that many of the included studies were randomized controlled trials, while just four of them were observational studies. Similarly, publication bias was low, as shown by the symmetric funnel plots.

Overall, the risk of developing cancer during therapy with tofacitinib was similar to that in patients treated with a placebo or other drugs (RR = 1.06; *p* = 0.57). Separate analyses limited to only studies with a placebo control and to those with biological therapy as an active control also showed no significant difference in the cancer risk. Only the analysis between tofacitinib and anti-TNF agents revealed a slightly increased overall risk of cancer in the tofacitinib groups (RR = 1.40 *p* = 0.02); this result was maintained when NMSC was excluded from the analysis (RR = 1.47; *p* = 0.03) and was lost in the analysis of NMSC alone (RR = 1.3; *p* = 0.88).

The fact that the risk of cancer was not the same for all the analyses performed in this study may be due to different study designs and populations. Moreover, even though 18 out of the 26 selected studies compared tofacitinib to a placebo, 10 of them were excluded from the meta-analysis because cancer was not detected. In contrast, four of the six studies that compared tofacitinib and anti-TNF agents detected at least one case of cancer. This difference may have affected the statistical analysis, leading to an overestimation of the tofacitinib-related cancer risk.

This study has some limitations. The fact that many RCTs were included could suggest that our findings are not representative of clinical practice. Importantly, the included studies were heterogeneous in terms of different IMIDs, baseline characteristics of the patients, sample size, tofacitinib dose and duration of treatment, and follow-up. Particularly, the tofacitinib dosage was very heterogeneous in trials of different phases and changed during the studies in some of the phase 3 trials, so we could not calculate different cancer risks associated with different dosages of tofacitinib. Regarding the follow up-time and sample size of the study, we tried to minimize these differences by expressing the follow-up time in units of person-months. In addition, some of the baseline characteristics of the patients could not be taken into account; first of all, the mean age of patients in different studies was not reported, as each study reported the mean age for each group of patients, but frequently, two or more groups of patients had to be considered altogether for the purpose of this metanalysis. In this case, a pooled analysis should have been carried out, but necessary data were not available for every study. Moreover, we do not know the smoking habits of the patients included in each study, and this could represent a confounding factor. Nonetheless, two major points of strength of our study are the inclusion of only studies with control groups and the consideration of all indications for tofacitinib.

Our finding of a slightly higher risk of cancer (excluding NMSC) in patients who took tofacitinib than in those treated with anti-TNF agents should be interpreted with caution. This result cannot be generalized to every age group and every IMID, because aging is an independent risk factor for cancer and IMIDs increase the risk of specific neoplasms [15]. Indeed, in our meta-analyses, the six studies that compared tofacitinib to anti-TNF agents all enrolled patients with a mean age around 50 years who had rheumatoid arthritis (with the exception of one study of psoriatic arthritis). Moreover, patients in the ORAL Surveillance study [19] were all over 50 years and had at least one major cardiovascular risk factor (e.g., smoking) that increases their neoplastic risk. In the rheumatoid arthritis population, malignancy is a leading cause of death [40,41], especially in patients not undergoing immunosuppressive therapy [42]. Nonetheless, this is also supported by our finding on RA indications, which is the only subgroup confirming the slightly increased risk of cancer in patients undergoing tofacitinib vs. anti-TNF. Our finding that the cancer risk under tofacitinib therapy was higher than under anti-TNF therapy but not for the placebo can be explained by the higher risk of cancer induced by uncontrolled inflammation in patients not being treated with immunosuppressive or biological drugs or by a potential direct antineoplastic effect of anti-TNF agents [15]

## 5. Conclusions

Overall, our results about the lack of an increased neoplastic risk associated with tofacitinib are reassuring. Further prospective studies, designed ad hoc in real clinical practice, are needed to better define the potential cancer risk of tofacitinib therapy and also of the whole anti-JAK class, controlling for confounders due to the different IMIDs, different baseline characteristics of the patients, and the impact of ongoing and previous immunomodulant and biologic therapies.

## Figures and Tables

**Table 1:** General characteristics of the studies reported in 22 articles selected for analysis.

Reference	Study Type	Cases (*n*)	Controls (*n*)	Follow-Up (months)	Tofacitinib Indication	Control Treatment
van der Heijde et al., 2019 [20]	Phase III trial	637	160	6	RA	Placebo
Fleischmann et al., 2017 [21]	Phase IIIb/IV trial	760	386	12	RA	Adalimumab
Lee et al., 2014 [22]	Phase III trial	770	186	24	RA	Methotrexate
Kremer et al., 2013 [23]	Phase III trial	636	159	6	RA	Placebo
van Vollenhoven et al., 2012 [24]	Phase III trial	405	312	6	RA	Placebo and adalimumab
Fleischmann et al., 2012 [25]	Phase III trial	488	122	3	RA	Placebo
Fleischmann et al., 2012 [26]	Phase Iib trial	272	112	3	RA	Placebo and adalimumab
Tanaka et al., 2015 [27]	Phase II trial	265	53	3	RA	Placebo
Kremer et al., 2012 [28]	Phase Iia trial	440	69	6	RA	Placebo
Tanaka et al., 2011 [29]	Phase II trial	108	28	3	RA	Placebo
Kremer et al., 2009 [30]	Phase Iia trial	199	65	2	RA	Placebo
Straatmijer et al., 2023 [31]	Observational study	152	150	12	UC	Vedolizumab
Hyun et al., 2022 [32]	Observational study	13	37	12	UC	Vedolizumab and Ustekinumab
Dalal et al., 2021 [33]	Observational study	45	36	13.6	UC	Ustekinumab
Sandborn et al., 2017 [2]	Phase III trials				UC	Placebo
OCTAVE Induction 1		492	122	2		
OCTAVE Induction 2		435	112	2		
OCTAVE Sustain		395	198	13		
Sandborn et al., 2012 [34]	Phase III trial	146	48	3	UC	Placebo
Ytterberg et al., 2022 [15]	Phase IV trial	2911	1451	38.5	RA	Anti-TNF drugs
Mease et al., 2017 [35]	Phase III trial				PsA	Placebo and adalimumab
Part A		211	105	3		
Part B		316	106	12		
Panés et al., 2017 [36]	Phase II trials				CD	Placebo
Induction study		172	91	2		
Maintenance study		121	59	5		
Deodhar et al., 2021 [37]	Phase II trial	133	136	4	AS	Placebo
van der Heijde et al., 2017 [38]	Phase II trial	156	51	4	AS	Placebo
Kremer et al., 2021 [39]	Observational study	1999	6354	12	RA	Biological drugs

RA, rheumatoid arthritis; UC, ulcerative colitis; PsA, psoriatic arthritis; CD, Crohn’s disease; AS, ankylosing spondylitis.

**Table 2:** Quality of the studies reported in the 22 selected articles, according to the Newcastle–Ottawa scale (NOS).

Reference	Selection	Comparability	Exposure	NOS Score
van der Heijde et al., 2019 [20]	4	1	3	8
Fleischmann et al., 2017 [21]	3	2	4	9
Lee et al., 2014 [22]	3	2	4	9
Kremer et al., 2014 [23]	4	1	3	8
van Vollenhoven et al., 2012 [24]	3	1	3	7
Fleischmann et al., 2012 [25]	4	2	2	8
Fleischmann et al., 2012 [26]	4	2	4	10
Tanaka et al., 2015 [27]	3	1	3	7
Kremer et al., 2012 [28]	4	2	4	10
Tanaka et al., 2011 [29]	4	2	2	8
Kremer et al., 2009 [30]	4	2	4	10
Straatmijer et al., 2023 [31]	3	2	3	8
Hyun et al., 2022 [32]	3	0	3	6
Dalal et al., 2021 [33]	3	0	3	6
Sandborn et al., 2017 [2]	4	1	4	9
Sandborn et al., 2012 [34]	4	2	4	10
Ytterberg et al., 2022 [15]	4	2	4	10
Mease et al., 2017 [35]	4	2	4	10
Panés et al., 2017 [36]	3	2	3	8
Deodhar et al., 2021 [37]	2	2	3	7
van der Heijde et al., 2017 [38]	3	2	3	8
Kremer et al., 2021 [39]	2	2	2	6

**Table 3:** Follow-up duration and numbers of tumors detected in the included studies, by treatment group.

Reference	Tofacitinib Treatment				Placebo or Active Control Treatment			
	Follow-Up (Person-mo.)	Cancer (excl. NMSC)	NMSC	Total Cancer	Follow-Up (Person-mo.)	Cancer (excl. NMSC)	NMSC	Total Cancer
van der Heijde et al. [20]	3822	4	4	8	960	0	1	1
Fleischmann et al. [21]	9120	1	2	3	4632	0	1	1
Lee et al. [22]	18,480	5	0	5	4464	1	0	1
Kremer et al. [23]	3816	0	0	0	954	0	0	0
van Vollenhoven et al. [24]	2430	3	0	3	1872	1	0	1
Fleischmann et al. [25]	1464	1	0	1	366	0	0	0
Fleischmann et al. [26]	816	0	0	0	336	0	0	0
Tanaka et al. [27]	795	0	0	0	159	0	0	0
Kremer et al. [28]	2640	0	0	0	414	0	0	0
Tanaka et al. [29]	324	0	0	0	84	0	0	0
Kremer et al. [30]	398	0	0	0	130	0	0	0
Straatmijer et al. [31]	1824	0	0	0	1800	0	0	0
Hyun et al. [32]	156	0	0	0	444	0	0	0
Dalal et al. [33]	612	0	0	0	490	0	0	0
OCTAVE Induction 1 [2]	984	0	1	1	244	0	0	0
OCTAVE Induction 2 [2]	870	0	1	1	224	0	0	0
OCTAVE Sustain [2]	5135	0	3	3	2574	1	1	2
Sandborn et al. [34]	438	0	0	0	144	0	0	0
Ytterberg et al. [15]	112,074	122	0	122	55,864	42	0	42
Mease et al. [35] Part A	633	2	1	3	315	0	0	0
Mease et al. [35] Part B	3792	3	1	4	1272	0	0	0
Panés et al. [36] Induction	344	1	0	1	182	0	1	1
Panés et al. [36] Maintenance	605	0	0	0	295	0	0	0
Deodhar et al. [37]	532	0	0	0	544	0	0	0
van der Heijde et al. [38]	624	0	0	0	204	0	0	0
Kremer et al. [39]	23,988	28	34	62	76,248	101	136	237

NMSC, non-melanoma skin cancer.

## Data Availability

All data have been collected in a database and are available upon request to corresponding author.

## Supplementary Materials

The following supporting information can be downloaded at: https://www.mdpi.com/article/10.3390/cancers15082197/s1, Figure S1: Cancer risk in patients with RA. A. Overall cancer risk in tofacitinib vs control (placebo + active treatment) random RR 1.04 (95% CI 0.84-1.29; I2 0%; *p* = 0.65). B. cancer risk without NMSC in tofacitinib vs control (placebo + active treatment) random RR 1.18 (95% CI 0.91–1.53; I2 0%; *p* = 0.20). C. NMSC risk in tofacitinib vs. control (placebo + active treatment) random RR 0.80 (95% CI 0.56–1.16; I2 0%; *p* = 0.20). D. Lung cancer risk in tofacitinib vs control (placebo + active treatment) random RR 0.94 (95% CI 0.43–2.07; I2 0%; *p* = 0.87); Figure S2: Cancer risk in RA patients treated with tofacitinib vs placebo (A), biological drugs (B) or anti TNF (C). A. Overall cancer risk in RA patients treated with tofacitinib vs placebo. Random effect RR 1.19 (95% CI 0.30–4.70; I2 0%; *p* = 0.80). B. Overall cancer risk in RA patients treated with tofacitinib vs all biological drugs. Random effect RR 1.04 (95% CI 0.84–1.29; I2 0%; *p* = 0.70). C. Overall cancer risk in RA patients treated with tofacitinib vs anti TNF. Random effect RR 1.43 (95% CI 1.03–2.04; I2 0%; *p* = 0.03); Figure S3: Overall Cancer Risk in SpA patients undergoing tofacitinib vs control group (placebo or other active treatment). Overall cancer risk in SpA patients treated with tofacitinib vs all biological drugs. Random effect RR 2.99 (95% CI 0.54–16.6; I2 0%; *p* = 0.20); Figure S4: Cancer risk in UC patients undergoing tofacitinib vs control group (placebo or other active treatment). A. Overall cancer risk in UC patients undergoing tofacitinib vs control group (placebo or other active treatment) random effect RR 0.75 (95% CI 0.18–3.07; I2 0%; *p* = 0.70). B. NMSC risk in UC patients undergoing tofacitinib vs control group (placebo or other active treatment) random effect RR 1.70 (95% CI 0.22–5.30; I2 0%; *p* = 0.90). C. Cancer risk (excluding NMSC) in UC patients undergoing tofacitinib vs placebo; random effect RR 0.76 (95% CI 0.19–3.07; I2 0%; *p* = 0.70). D. NMSC risk in UC patients undergoing tofacitinib vs placebo; random effect RR 1.09 (95% CI 0.23-5.35; I2 0%; *p* = 0.94).
