# Supplementary material for: Cancer Risk in Patients Treated with the JAK Inhibitor Tofacitinib: Systematic Review and Meta-Analysis
Source: Cancers (Basel). 2023 Apr 7;15(8):2197. doi: 10.3390/cancers15082197 (PMC10136459; doi:10.3390/cancers15082197)
Supplement: Supplementary file 1 [file cancers-15-02197-s001.zip › cancers-2283562-supplementary.pdf]

SUPPLEMENTARY MATERIALS

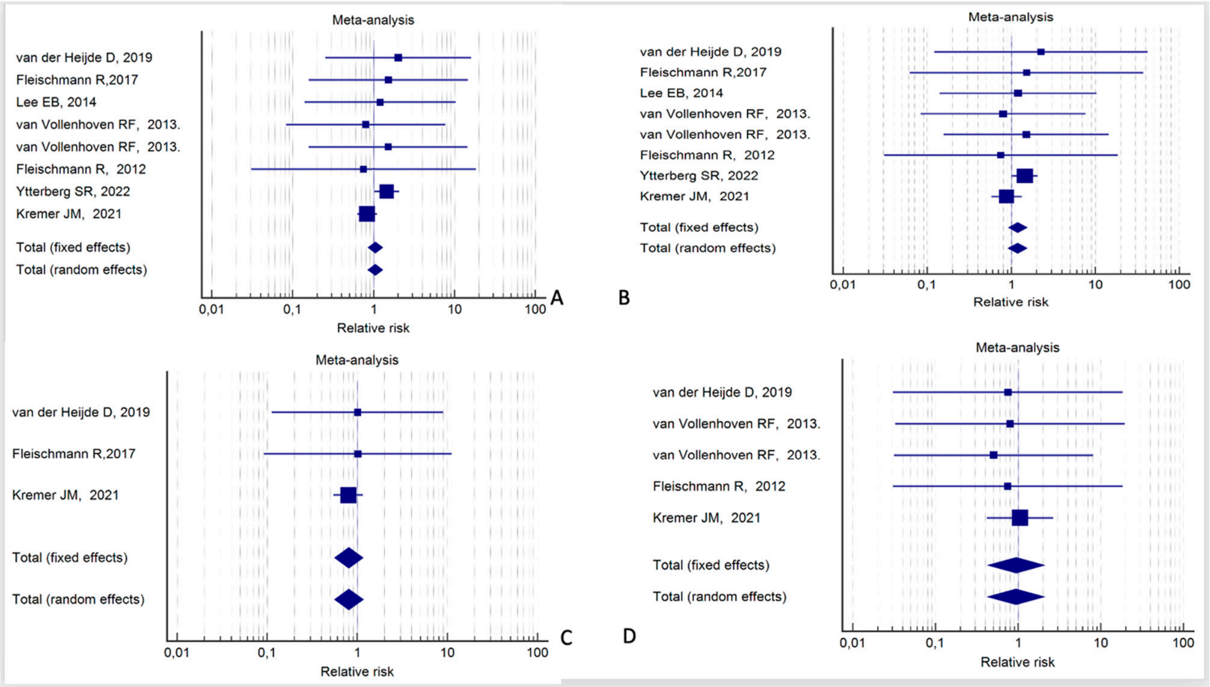

Figure S1. Cancer risk in patients with RA. **A.** Overall cancer risk in tofacitinib vs control (placebo + active treatment) random RR 1.04 (95% CI 0.84-1.29; I^2 0%; p=0.65). **B**. cancer risk without NMSC in tofacitinib vs control (placebo + active treatment) random RR 1.18 (95% CI 0.91-1.53; I^2 0%; p=0.20). **C**. NMSC risk in tofacitinib vs control (placebo + active treatment) random RR 0.80 (95% CI 0.56-1.16; I^2 0%; p=0.20). **D**. Lung cancer risk in tofacitinib vs control (placebo + active treatment) random RR 0.94 (95% CI 0.43-2.07; I^2 0%; p=0.87). [2–7,9]

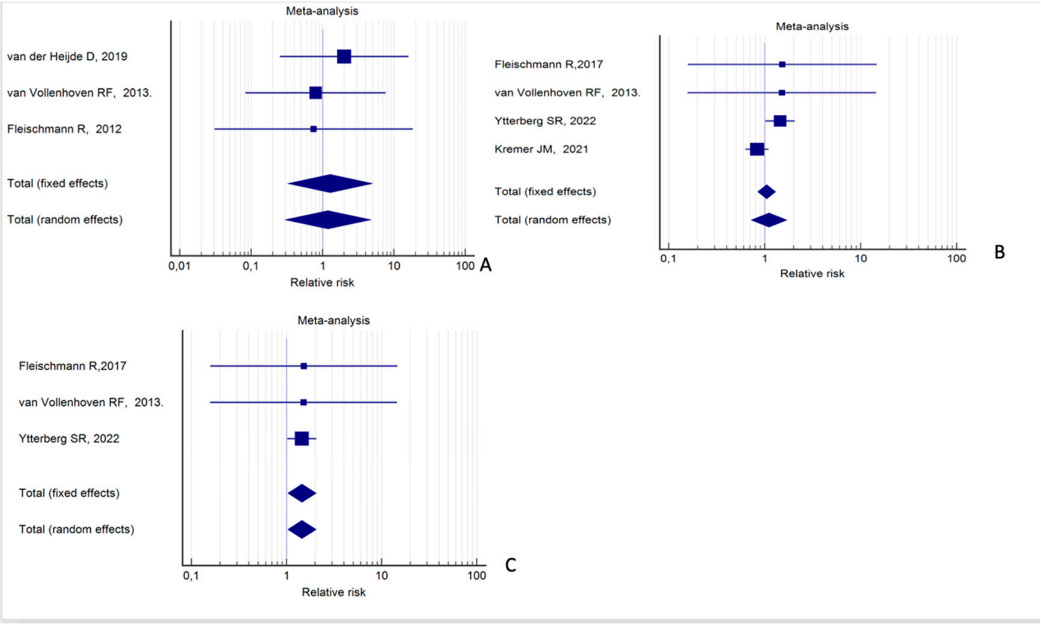

Figure S2. Cancer risk in RA patients treated with tofacitinib vs placebo (A), biological drugs (B) or anti TNF (C). A. Overall cancer risk in RA patients treated with tofacitinib vs placebo. Random effect RR 1.19 (95% CI 0.30-4.70; I^2 0%; p= 0.80). B. Overall cancer risk in RA patients treated with tofacitinib vs all biological drugs. Random effect RR 1.04 (95% CI 0.84-1.29; I^2 0%; p= 0.70). C. Overall cancer risk in RA patients treated with tofacitinib vs anti TNF. Random effect RR 1.43 (95% CI 1.03-2.04; I^2 0%; p= 0.03). [2–4,6,7,9]

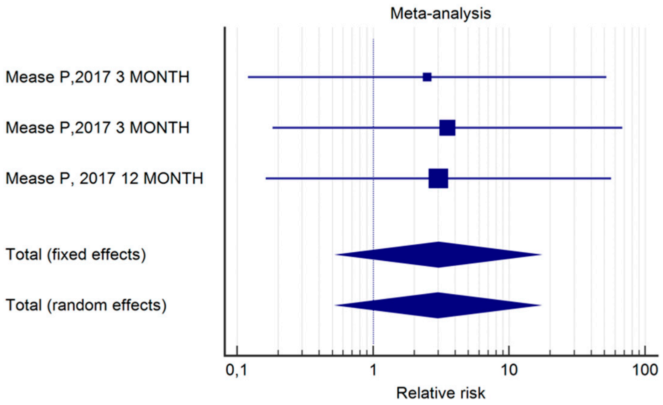

Figure S3. Overall Cancer Risk in SpA patients undergoing tofacitinib vs control group (placebo or other active treatment). Overall cancer risk in SpA patients treated with tofacitinib vs all biological drugs. Random effect RR 2.99 (95% CI 0.54-16.6; I^2 0%; p= 0.20). [8]

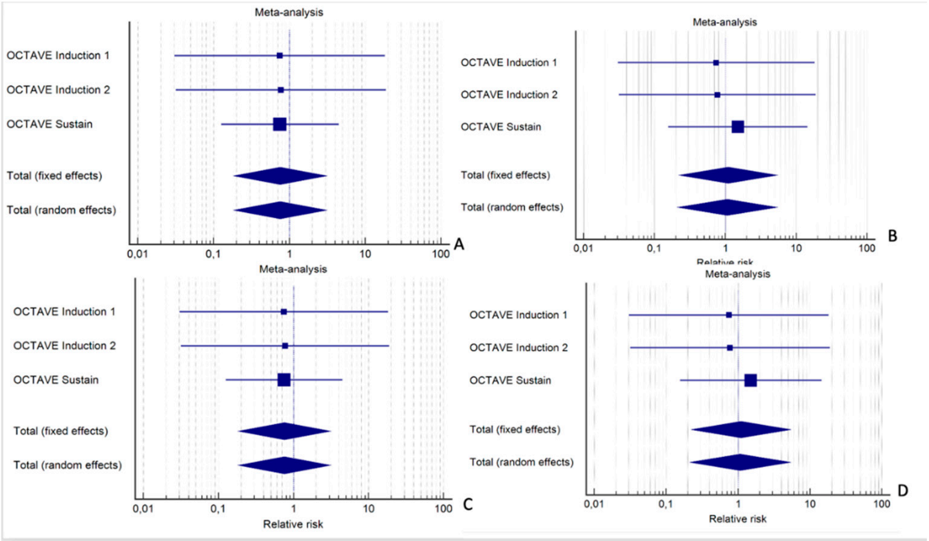

Figure S4. Cancer risk in UC patients undergoing tofacitinib vs control group (placebo or other active treatment). **A.** Overall cancer risk in UC patients undergoing tofacitinib vs control group (placebo or other active treatment) random effect RR 0.75 (95% CI 0.18-3.07; I^2 0%; p= 0.70). **B**. NMSC risk in UC patients undergoing tofacitinib vs control group (placebo or other active treatment) random effect RR 1.70 (95% CI 0.22-5.30; I^2 0%; p= 0.90). **C**. Cancer risk (excluding NMSC) in UC patients undergoing tofacitinib vs placebo; random effect RR 0.76 (95% CI 0.19-3.07; I^2 0%; p= 0.70). **D.** NMSC risk in UC patients undergoing tofacitinib vs placebo; random effect RR 1.09 (95% CI 0.23-5.35; I^2 0%; p= 0.94) [1]

References

1. Dai, C.; Jiang, M.; Sun, M.-J. Tofacitinib as Induction and Maintenance Therapy for Ulcerative Colitis. *N. Engl. J. Med.* **2017**, *377*, 496. https://doi.org/10.1056/NEJMc1707500.
2. Ytterberg, S.R.; Bhatt, D.L.; Mikuls, T.R.; Koch, G.G.; Fleischmann, R.; Rivas, J.L.; Germino, R.; Menon, S.; Sun, Y.; Wang, C.; et al. Cardiovascular and Cancer Risk with Tofacitinib in Rheumatoid Arthritis. *N. Engl. J. Med.* **2022**, *386*, 316–326. https://doi.org/10.1056/NEJMOA2109927.
3. van der Heijde, D.; Strand, V.; Tanaka, Y.; Keystone, E.; Kremer, J.; Zerbini, C.A.F.; Cardiel, M.H.; Cohen, S.; Nash, P.; Song, Y.W.; et al. Tofacitinib in Combination With Methotrexate in Patients With Rheumatoid Arthritis: Clinical Efficacy, Radiographic, and Safety Outcomes From a Twenty-Four-Month, Phase III Study. *Arthritis Rheumatol.* **2019**, *71*, 878–891. https://doi.org/10.1002/ART.40803.
4. Fleischmann, R.; Mysler, E.; Hall, S.; Kivitz, A.J.; Moots, R.J.; Luo, Z.; DeMasi, R.; Soma, K.; Zhang, R.; Takiya, L.; et al. Efficacy and safety of tofacitinib monotherapy, tofacitinib with methotrexate, and adalimumab with methotrexate in patients with rheumatoid arthritis (ORAL Strategy): A phase 3b/4, double-blind, head-to-head, randomised controlled trial. *Lancet* **2017**, *390*, 457–468. https://doi.org/10.1016/S0140-6736(17)31618-5.
5. Lee, E.B.; Fleischmann, R.; Hall, S.; Wilkinson, B.; Bradley, J.D.; Gruben, D.; Koncz, T.; Krishnaswami, S.; Wallenstein, G.V.; Zang, C.; et al. Tofacitinib versus methotrexate in rheumatoid arthritis. *N. Engl. J. Med.* **2014**, *370*, 2377–2386. https://doi.org/10.1056/NEJMOA1310476.
6. van Vollenhoven, R.F.; Fleischmann, R.; Cohen, S.; Lee, E.B.; García Meijide, J.A.; Wagner, S.; Forejtova, S.; Zwillich, S.H.; Gruben, D.; Koncz, T.; et al. Tofacitinib or adalimumab versus placebo in rheumatoid arthritis. *N. Engl. J. Med.* **2012**, *367*, 508–519. https://doi.org/10.1056/NEJMOA1112072.
7. Fleischmann, R.; Kremer, J.; Cush, J.; Schulze-Koops, H.; Connell, C.A.; Bradley, J.D.; Gruben, D.; Wallenstein, G.V.; Zwillich, S.H.; Kanik, K.S. Placebo-controlled trial of tofacitinib monotherapy in rheumatoid arthritis. *N. Engl. J. Med.* **2012**, *367*, 495–507. https://doi.org/10.1056/NEJMOA1109071.
8. Mease, P.; Hall, S.; FitzGerald, O.; van der Heijde, D.; Merola, J.F.; Avila-Zapata, F.; Cieślak, D.; Graham, D.; Wang, C.; Menon, S.; et al. Tofacitinib or Adalimumab versus Placebo for Psoriatic Arthritis. *N. Engl. J. Med.* **2017**, *377*, 1537–1550. https://doi.org/10.1056/NEJMOA1615975.
9. Kremer, J.M.; Bingham, C.O.; Cappelli, L.C.; Greenberg, J.D.; Madsen, A.M.; Geier, J.; Rivas, J.L.; Onofrei, A.M.; Barr, C.J.; Pappas, D.A.; et al. Postapproval Comparative Safety Study of Tofacitinib and Biological Disease-Modifying Antirheumatic Drugs: 5-Year Results from a United States-Based Rheumatoid Arthritis Registry. *ACR Open Rheumatol.* **2021**, *3*, 173–184. https://doi.org/10.1002/ACR2.11232.
